# Small Structural Proteins E and M Render the SARS-CoV-2 Pseudovirus More Infectious and Reveal the Phenotype of Natural Viral Variants

**DOI:** 10.3390/ijms22169087

**Published:** 2021-08-23

**Authors:** Hsin-I Wang, Zih-Shiuan Chuang, Yu-Ting Kao, Yi-Ling Lin, Jian-Jong Liang, Chun-Che Liao, Ching-Len Liao, Michael M. C. Lai, Chia-Yi Yu

**Affiliations:** 1 National Institute of Infectious Diseases and Vaccinology, National Health Research Institutes, Miaoli 350, Taiwan; 070212@nhri.org.tw (H.-I.W.); 080945@nhri.org.tw (Z.-S.C.); 070207@nhri.org.tw (Y.-T.K.); chinglen@nhri.org.tw (C.-L.L.); 2 Institute of Biomedical Sciences, Academia Sinica, Taipei 115, Taiwan; yll@ibms.sinica.edu.tw (Y.-L.L.); jjliang1234@yahoo.com.tw (J.-J.L.); jfliao@ibms.sinica.edu.tw (C.-C.L.); 3 Biomedical Translation Research Center, Academia Sinica, Taipei 115, Taiwan; 4 Research Center for Emerging Viruses, China Medical University Hospital, Taichung 404, Taiwan; 5 Institute of Molecular Biology, Academia Sinica, Taipei 115, Taiwan

**Keywords:** SARS-CoV-2, structural protein, E and M proteins, pseudovirus

## Abstract

The SARS-CoV-2 pseudovirus is a commonly used strategy that mimics certain biological functions of the authentic virus by relying on biological legitimacy at the molecular level. Despite the fact that spike (S), envelope (E), and membrane (M) proteins together wrap up the SARS-CoV-2 virion, most of the reported pseudotype viruses consist of only the S protein. Here, we report that the presence of E and M increased the virion infectivity by promoting the S protein priming. The S, E, and M (SEM)-coated pseudovirion is spherical, containing crown-like spikes on the surface. Both S and SEM pseudoviruses packaged the same amounts of viral RNA, but the SEM virus bound more efficiently to cells stably expressing the viral receptor human angiotensin-converting enzyme II (hACE2) and became more infectious. Using this SEM pseudovirus, we examined the infectivity and antigenic properties of the natural SARS-CoV-2 variants. We showed that some variants have higher infectivity than the original virus and that some render the neutralizing plasma with lower potency. These studies thus revealed possible mechanisms of the dissemination advantage of these variants. Hence, the SEM pseudovirion provides a useful tool to evaluate the viral infectivity and capability of convalescent sera in neutralizing specific SARS-CoV-2 S dominant variants.

## 1. Introduction

The current outbreak of human coronavirus disease of 2019 (COVID-19) is attributed to the severe acute respiratory syndrome coronavirus 2 (SARS-CoV-2) infection [1]. The virus undergoes rapid mutation [2] so that there is a strong need to examine the viral genome or antigenicity of SARS-CoV-2 and its variants frequently. An approach functionally evaluating virus-binding or entry without the virus replication will significantly reduce the risk of biosafety concern. The pseudotyped virion is a well-known virology approach wrapping a virus in non-self-structural proteins on demand for the aforementioned purpose. However, using differently prepared pseudovirions may vary the experimental conclusions.

All the coronavirus virions contain at least four structural proteins: spike (S), envelope (E), membrane (M), and nucleocapsid (N) proteins [3]. The N protein forms a ribonucleoprotein complex (RNP) by associating with the viral genome [4], whereas the S, E, and M proteins packed the RNP in the endoplasmic reticulum/Golgi intermediate compartment (ER/GIC) to form the virion egressed by exocytosis. The virion assembly requires E and M proteins [5], and the S protein trimers contribute to the crown-like morphology of the virion [6]. The S protein comprises N-terminal S1 and C-terminal S2 subunits responsible for receptor binding and virus-host membrane fusion, respectively. The S1 receptor-binding domain (RBD) determines cell tropism by recognizing a specific receptor on the cell surface, e.g., the human angiotensin-converting enzyme II (hACE2) [7,8].

The fusion of viral envelope and host membrane is mediated by the fusion peptide (FP) domain in the S2. The FP domain is hidden until a protease-mediated cleavage at the S1/S2 junction or an alternative site close to the N-terminus of FP domain (S2′ site), which is called the S protein priming [9]. The timing of coronavirus S protein priming governs virus entry because it would be abortive if the priming is blocked or occurs before S reaches its host receptor [9]. In the case of SARS-CoV infection, the S could be primed by a type II transmembrane serine protease TMPRSS2 at the surface of the target cells [10] or by the cathepsin L in the endosome [11]. Although SARS-CoV and SARS-CoV-2 share a similar strategy in the S protein priming, the S protein of SARS-CoV-2 contains an additional furin cleavage site between RBD and FP [8].

The lentiviral vector is a well-established transduction system that wraps recombinant human immunodeficiency virus (HIV) genome harboring genes of interest inside the envelope consisting of the vesicular stomatitis virus (VSV) structural envelope G (VSV G) [12]. In the case of SARS-CoV-2 S, an additional step of protein deletion is required for sufficient pseudovirion infectivity [13,14] because the lentivirus buds from the plasma membrane, not the ER membrane. Here, we showed that the E and M proteins enhance the SARS-CoV-2 pseudovirion infectivity and promote S protein priming on the virion that can accurately reflect the properties of SARS-CoV-2 and its genetic variants.

## 2. Results

### 2.1. SARS-CoV-2 Structural Proteins E and M Enhance S Priming

**Figure 1:**
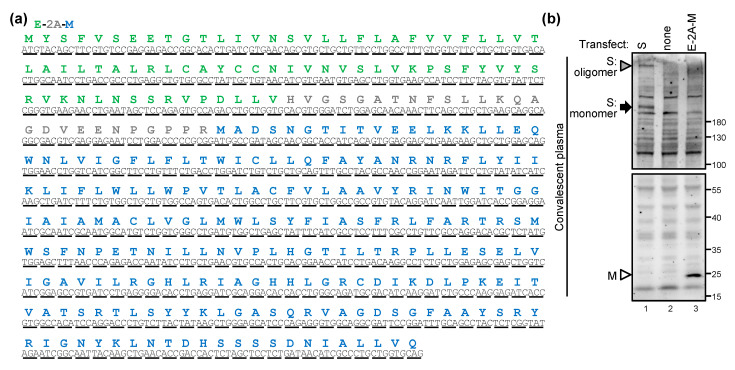
Protein expression of the SARS-CoV-2 structural proteins designed: (**a**) the sequence of the synthesized E-2A-M. (**b**) BHK-21 cells were transfected with SARS-CoV-2 S or E-2A-M as indicated, and the cell lysates were analyzed by Western blot (WB) using the convalescent human plasma. Black arrow, S monomer; gray triangle, S oligomer; white triangle, M.

The authentic SARS-CoV-2 virion is enveloped with S, E, and M proteins. In contrast, most of the SARS-CoV-2 pseudovirion preparations contain only S. To explore the functions of SARS-CoV-2 E and M proteins, a bicistronic mammalian expression cassette coexpressing the E and M by introducing a picornavirus 2A protein cleavage sequence [15,16] (E-2A-M) was designed and codon-optimized (Figure 1a). None of the proteins were tagged with an extraneous sequence to avoid unnecessary effects. The S and M proteins were detected in the cells transfected with S and E-2A-M, respectively, by the convalescent plasma recovered from COVID-19 (Figure 1b). The S protein was present as two protein species, representing the full-length S protein in monomer (180–200 kDa) and oligomer (likely trimer) forms [17]. The M protein has a molecular weight of approximately 25 kDa as predicted, whereas E protein was not detected, probably due to poor antigenicity.

**Figure 2:**
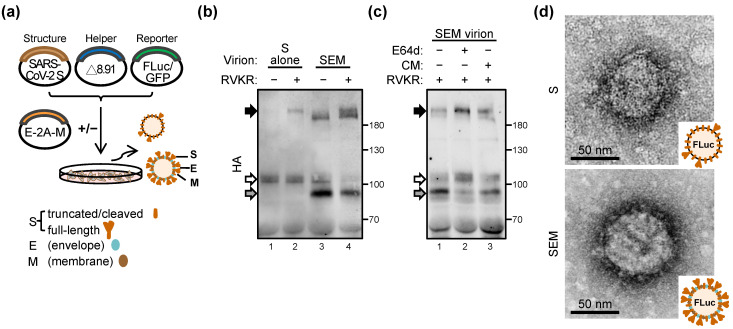
In *trans*, supplying SARS-CoV-2 E and M proteins promotes the pseudovirion S priming and contributes crown-like morphology: (**a**) schematic illustration of the pseudovirion preparation. Plasmids of SARS-CoV-2 S protein plus lenti-helper and reporter (FLuc, *firefly luciferase*; GFP, green fluorescent protein) were transfected into 293T/17 cells with or without the E-2A-M construct. (**b**,**c**) The pseudovirions made by different preparations were analyzed by WB using the anti-HA antibody. The presence (+) or absence (−) of the inhibitors in each preparation are indicated. The SARS-CoV-2 S protein is C-terminal HA-tagged. RVKR, furin inhibitor (10 μM); E64d, cathepsin L inhibitor (100 μM); camostat mesylate (CM), TMPRSS2 inhibitor (250 μM). Black arrow, full-length S; white arrow, furin-cleaved S; gray arrow, primed S2′. (**d**) Transmission electron microscopy (TEM) analysis of the pseudovirion coated with S alone (S) or with S, E and M (SEM).

We then prepared the lentiviral vector-based pseudovirus coated by the S protein together with (termed SEM pseudovirus) or without (S-alone) E-2A-M (Figure 2a). The virion protein analysis showed that a very faint band corresponding to the full-length S protein (Figure 2b, black arrow) was detected in the S-alone pseudovirion (Figure 2b, lane 1). This protein was significantly increased in the presence of RVKR, the furin protease inhibitor (Figure 2b, lane 2). Unexpectedly, SEM virion harbors an S protein of smaller size, which most likely represents the S priming product (Figure 2b, lane 3; gray arrow) in addition to the furin-cleaved S (Figure 2b, lane 3 vs. 4; white arrow). The furin-resistant S priming product of the SEM virion seemed likely mediated by the E64d-sensitive cathepsins rather than the camostat mesylate (CM)-sensitive TMPRSS2 protease (Figure 2c, lane 2; gray arrow). Consequently, the different virion processes led the SEM virion and S-alone virion to undergo different S-priming processes. Significantly, crown-like spikes can be seen on the virion surface of the SEM but not the S-virion (Figure 2d). In contrast to the authentic SARS-CoV-2 virion with 120–160 nm in diameter [1], both the S and SEM are smaller (~80 nm in diameter).

### 2.2. The E and M Proteins Make SARS-CoV-2 Pseudovirus Highly Infectious

**Figure 3:**
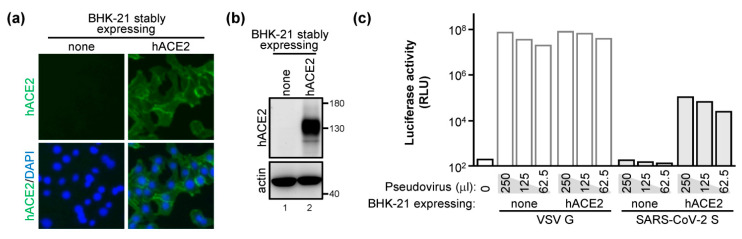
SARS-CoV-2 S-pseudotyped virus infects the BHK-hACE2 cells: (**a**,**b**) BHK-21 cells stably expressing hACE2 were fixed without permeabilization and then analyzed by immunofluorescence assay (IFA) (**a**) or were analyzed by WB (**b**) with the indicated antibodies. (**c**) Parental or hACE2-expressing BHK-21 cells were infected with FLuc reporter-carrying viruses pseudotyped with the VSV G or SARS-CoV-2 S protein as indicated. The luciferase activity was measured at 48 h post-infection and represented as the infectivity. RLU, relative light unit.

To specifically evaluate the infectivity of SARS-CoV-2 pseudovirions, we stably expressed hACE2 into hamster cell line BHK-21 (BHK-hACE2) (Figure 3a,b). As a control, the VSV G protein-coated pseudovirus infected both parental and hACE2-expressing BHK-21 cells equally well (Figure 3c). Consistent with most previous studies, the SARS-CoV-2 S-alone pseudovirus could not infect the BHK-21 cells but successfully infected BHK-hACE2 cells in a dose-dependent manner (Figure 3c).

**Figure 4:**
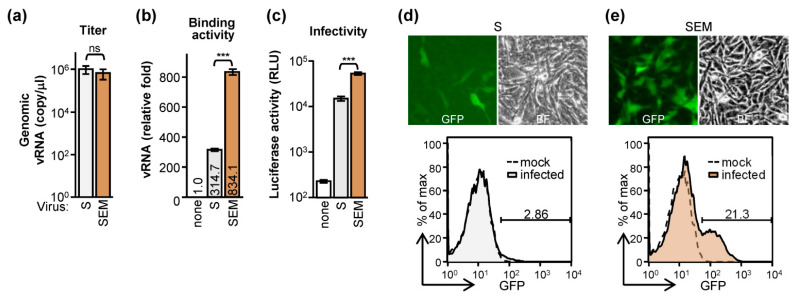
SARS-CoV-2 E and M proteins elevate the pseudovirion infectivity: (**a**) the S-alone and SEM pseudovirus titer were measured by qRT-PCR targeting genomic RNA of the virion. (**b**) BHK-hACE2 cells were incubated with S-alone or SEM pseudovirus at 4 °C for 3 h and then washed. The virus-binding activity was estimated by analyzing genomic RNA of the virion bound to the cell surface. (**c**) BHK-hACE2 cells were infected with S-alone or SEM-pseudovirus harboring FLuc reporter. The luciferase activity was measured at 48 h post-infection. Data (**a**–**c**) are mean ± SD, *n* = 3 per group, and were compared by two-tailed Student’s *t* test. ns, no significance; ***, *p* < 0.001. (**d**,**e**) BHK-hACE2 cells were infected with the S-alone (**d**) or SEM (**e**) pseudovirus harboring GFP reporter as indicated. The infected cells were revealed by fluorescence microscopy (upper panel) and flow cytometry (lower panel).

Despite the fact that S-alone and SEM virus propagated equivalently in titer (Figure 4a), the virion binding assay showed that SEM virus binds more than S-alone virus to the BHK-hACE2 cells (Figure 4b). Thus, the SEM virus harboring *firefly luciferase* indeed was more infectious than the S-alone virus (Figure 4c). By infecting BHK-hACE2 cells with S- or SEM-pseudotyped viruses harboring GFP, we also found that the SEM virus significantly infected more cells (21.3% vs. 2.86%; Figure 4d,e). Thus, the E and M proteins enhance the SARS-CoV-2 pseudovirus infectivity.

### 2.3. The SEM Pseudovirus Is Highly Sensitive in the Bioassay Evaluating SARS-CoV-2 Neutralizing Antibodies

**Figure 5:**
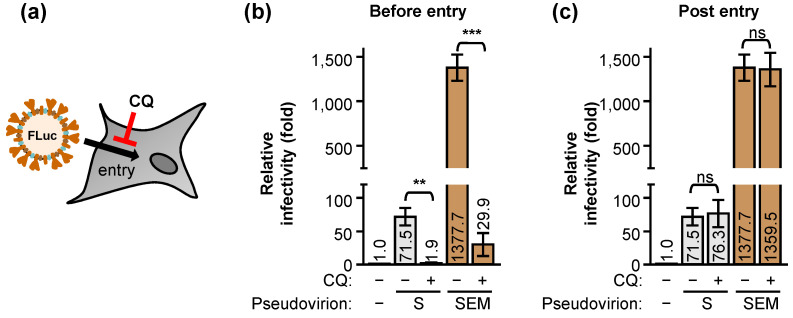
Chloroquine (CQ) suppresses the entry step of both S-alone and SEM pseudoviruses: (**a**) Chloroquine (CQ) known for suppressing virus entry was illustrated. (**b**,**c**) BHK-hACE2 cells were infected with the S-alone or SEM pseudovirus in the presence (+) or absence (−) of CQ (100 μM) as indicated. The cells were treated with CQ for 30 min before the infection (**b**; before-entry) or after 6 h of the infection (**c**; post-entry). The relative infectivity was estimated by the luciferase activity normalized to the mock group. Data (**b**,**c**) are mean ± SD, *n* = 3 per group, and were compared by two-tailed Student’s *t* test. **, *p* < 0.01; ***, *p* < 0.001; ns, no significance.

The above experiment shows that the SEM pseudotype virus binds to cells more strongly and has higher infectivity than the S pseudotype virus. We then asked whether this difference can contribute to the effects on virus entry. We used chloroquine (CQ), a compound with diprotic bases de-acidifying acidic organelles, to evaluate the pseudovirus applications in mimicking the entry studies of SARS-CoV-2 (Figure 5a). Both S- and SEM-pseudotyped viruses were sensitive to CQ treatment, when CQ was added before- (Figure 5b), but not post-entry, of the cells (Figure 5c). These data suggested that the S- and SEM-pseudotyped viruses share similar features of virus entry but not for a simple CQ therapy against any viral infection.

**Figure 6:**
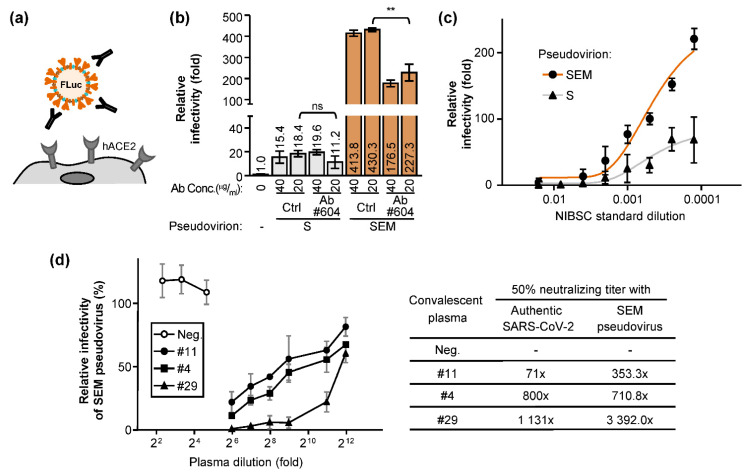
The SEM pseudovirion provides a better assessment window than the S-alone virus in studying SARS-CoV-2 virion entry: (**a**) the pseudovirus neutralization assay is shown in a diagram. (**b**,**c**) The monoclonal antibody (Ab#604) (**b**) or the standard plasma from the National Institute for Biological Standards and Control (NIBSC) (**c**) were diluted and incubated with pseudovirus for 1 h as indicated. The mixture was then transferred to infect BHK-hACE2 cells for 6 h. Samples were harvested at 48 h post-infection for luciferase activity. Data are mean ± SD (*n* = 3 per group). (**d**) Three Taiwanese convalescent plasma were two-fold serially diluted to neutralize the SEM-pseudovirus. The 50% neutralizing titer (NT50) of each sample determined by SEM pseudovirus or by authentic SARS-CoV-2 were summarized (right). Data (**b**–**d**) are mean ± SD, *n* = 3 per group, and were compared by two-tailed Student’s *t* test. ns, no significance; **, *p* < 0.01.

We also set up a neutralization assay (Figure 6a) assessing the capability of various antisera to block the infection of SARS-CoV-2, its mutants, and natural variants of concern. Interestingly, while the monoclonal antibody (Ab#604) neutralized the SEM virus infection, the same antibody with equivalent concentration failed to neutralize the S-alone pseudovirus (Figure 6b). To further compare the SEM- with S-pseudoviruses credibly, we neutralized these two pseudoviruses with different dilutions of the WHO international standard plasma (pooled from eleven individuals recovered from SARS-CoV-2 infection from the National Institute for Biological Standards and Control (NIBSC)). We found that the SEM virus was better than the S-alone virus in this neutralization assay (Figure 6c), probably because of the higher virus infectivity and/or because the SEM virus allosterically displays more epitopes targeted by the neutralizing antibodies.

Since the SEM virus is superior to the S-alone virus in the neutralization assay, we asked whether the result using the SEM virus correlates with the data using authentic SARS-CoV-2. Three convalescent plasmas with low (Tw#11), medium (Tw#4), and high (Tw#29) neutralizing titers were measured by using the authentic SARS-CoV-2. The identical plasma samples were single-blinded and subjected to be titrated again by using the SEM pseudovirus (Figure 6d). Regardless of the neutralization titers of the three different plasma, they yielded the same neutralization trends (Figure 6d), showing that the SEM pseudotype virus reflected accurately the properties of the natural SARS-CoV-2 in their neutralizing capability.

### 2.4. The SEM Pseudoviruses of Some Natural S Variants Showed the Capability to Resist Neutralizing Antibodies

**Figure 7:**
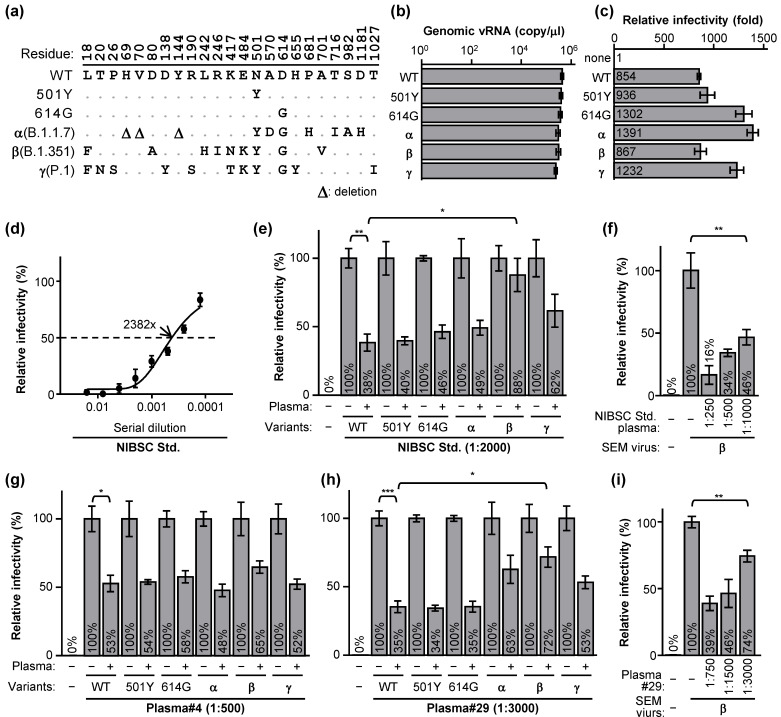
The SEM pseudovirion infectivity and convalescent plasma resistance vary with the S variants: (**a**) the indicated protein residues of the S variants for pseudotyping the SEM viruses were aligned. Variants: α, B.1.1.7; β, B.1.351; γ, P.1. (**b**) Each SEM pseudovirus preparation with the indicated S variant was titered by quantifying the genomic RNA of the virion. (**c**) The relative infectivity was measured by the luciferase activity in contrast to the mock infection group. (**d**) The NT50 of the NIBSC standard plasma against the WT SEM pseudovirus was calculated as 2382x by Prism 6. (**e**) The neutralization assay of each SEM virus in the presence (+) or absence (−) of the 2000-fold diluted NIBSC plasma. (**f**) The SEM virus harboring the β S haplotype was neutralized by the serially diluted NIBSC plasma. (**g**,**h**) The two Taiwanese human plasma #4 (**g**) and #29 (**h**) were diluted to neutralize each SEM virus as indicated. (**i**) Same as panel f but in the presence of the diluted #29 plasma as indicated. Data are mean ± SD, *n* = 3 per group, and were compared by two-tailed Student’s *t* test. *, *p* < 0.05; **, *p* < 0.01; ***, *p* < 0.001.

We then used the SEM pseudotype virus to examine SARS-CoV-2 and its natural variants, which differ in their S sequence. We wrapped the SEM viruses with some dominant SARS-CoV-2 S variants (Figure 7a). The virion packaging efficiency seemed similar among these SEM viruses (Figure 7b), but the infectivity varied by the different S haplotypes (Figure 7c). As compared to the wild-type (WT), the 614G variants showed greatly enhanced infectivity (Figure 7c, WT vs. 501Y and 614G). Unlike the U.K. dominant α (B.1.1.7) or Brazil dominant γ (P.1) variants with enhanced infectivity, the infectivity of the South Africa dominant β (B.1.351) variant surprisingly remained similar to the WT (Figure 7c). To understand whether the convalescent plasma resistance contributes to the β variant dominancy, the NIBSC convalescent plasma from the U.K. was titrated by the WT SEM virus for 50% neutralization (NT50) titer (Figure 7d; 2382 folds of dilution). We then compared the relative infectivity of the SEM viruses with each S haplotype by percentage (mock infection as 0%) in the absence (100%) or presence of the 1:2000 diluted NIBSC plasma. At this concentration, diluted plasma neutralized the WT and most of the S variant viruses, except the β variant showing resistance to the U.K. NIBSC plasma (Figure 7e). Nevertheless, with the higher NIBSC plasma concentration, the β variant was neutralized (Figure 7f), indicating that the β variant is more resistant to neutralization by the pooled U.K. convalescent (NIBSC) sera. Thus, we checked the two Taiwanese convalescent plasmas (Tw#4 and Tw#29) for the capability to neutralize each S variant virus. While Tw#4 plasma neutralized all the listed variants equivalently (Figure 7g), the α, β, and γ showed marginally resistance to the Tw#29 plasma (Figure 7h,i). Thus, the infectivity and convalescent plasma resistance might together drive SARS-CoV-2 to evolve the various S variants worldwide.

## 3. Discussion

Studying SARS-CoV-2 entry remains important, especially when people will be vaccinated worldwide. Addressing the biosafety concerns handling the authentic virus, the SARS-CoV-2-like alternatives have been used for drug or antibody tests. Instead of the well-known needs of S mutation/deletion at the C-terminus for pseudovirus infectivity [13,14], we made a highly infectious SARS-CoV-2 pseudovirus by providing the neglected E and M structural proteins in *trans* that facilitates S-protein priming. The SEM pseudovirus preserves the S protein sequence integrity on-demand in studying the entry of all the known SARS-CoV-2 S variants [18] theoretically, including the alterations found very close to the C-terminus end [19]. Despite being smaller than the authentic SARS-CoV-2 in size, the SEM pseudovirus morphologically and functionally meets the requirement superior to the traditional S-alone virus in assessing neutralizing antibodies or compounds blocking SARS-CoV-2 entry.

The ubiquitous protease furin may reduce the pseudovirion infectivity by an untimely cleavage in the virus-packaging cells. The RVKR inhibitor suppressed S cleavage by furin but not the priming protease, most likely the cathepsin L, making the pseudovirion harbor both full-length and primed S proteins when the E and M are incorporated. Both the cleaved and full-length S proteins were found at the authentic SARS-CoV-2 virion [20], which functions differently. Without the full-length S-containing RBD, the virion failed to target host cells. In the circumstance of S-priming failure before entry, the yet-cleaved/primed S at the virion could take over to function at the next step of membrane fusion on the target cell surface. This scenario is also supported by the observation that the trypsin protease treatment could abrogate S-driven cell entry of the cell-free, but not cell-bound, viruses [11].

The crown structure of a coronavirus is attributed to the ~20 nm in length S homotrimer protruding on the virion surface [6,21]. Not all the S proteins on the SARS-CoV-2 virion are full-length, suggesting that a loose distribution of full-length S on the virion [21,22] could leave ample room for interacting host factors contributing to the virus entry. That might be the case for SARS-CoV-2 virion decorated with E and M proteins. The small integral E and M proteins at the virion membrane surface might allosterically spare space, allowing proteases to access the cleavage site priming S protein. The E and M might preferentially recruit proteases priming S, which could crowd out space for furin to cleave S. Despite not yet understanding how these proteins functionally contribute to the virus priming and infectivity in detail, the long-ignored E and M proteins should be considered to refine the SARS-CoV-2 pseudovirus-related bioassays currently.

Since the pseudovirus could never be the authentic ones, a pseudovirus system may or may not be good enough to accurately reflect the properties of the authentic virus. SARS-CoV-2 changes every time upon replication to adapt to the host cell. Every ribonucleotide or amino acid alteration beyond the S protein could contribute to its pathogenesis. The results from SEM virus infection here were solely affected by the SARS-CoV-2 entry step but no other infection processes, and vice versa. The pandemic of β variant harboring equivalent entry/infectivity with the WT might result from convalescent plasma resistance [23] and/or the ignored NS protein alterations beneficial to virus dissemination. The marginal but statistically significant convalescent plasma resistance of some SEM variants did not mean a new SARS-CoV-2 serotype but suggests a thorny problem if herd immunity against SARS-CoV-2 failed, regardless by vaccination or by natural infection. How a pseudovirus is similar enough to stand for the SARS-CoV-2 remains to be standardized. Until then, the SEM virus here is suitable to be allocated worldwide for mimicking SARS-CoV-2 and its growing S variants in virus entry studies and neutralization tests at low cost.

## 4. Materials and Methods

### 4.1. Inhibitors, Primary Antibodies, and Convalescent Plasma

The furin convertase inhibitor RVKR-cmk (ALX-260-022-M005) was from Enzo Life Sciences. The endosomal cysteine protease cathepsin B and L inhibitor E64d (E8640), the serine protease TMPRSS2 inhibitor camostat mesylate (CM; SML0057), and chloroquine (CQ; C6628) were from Sigma-Aldrich. Antibodies against HA (#3724S) and actin (NB600-501) were from Cell Signaling (Beverly, MA, USA) and Novus Biologicals (Centennial, CO, USA). The anti-hACE2 (GTX01160), the monoclonal antibody Ab#604, and the isotype control IgG antibody (GTX35009) were from GeneTex (Irvine, CA, USA). The WHO International Standard for anti-SARS-CoV-2 immunoglobulin was from the National Institute for Biological Standards and Control (NIBSC code: 20/136).

### 4.2. Plasmid Constructs

The lentiviral vector pLKO_AS3w.bsd was from the National RNAi Core Facility (Academia Sinica, Taipei, Taiwan). The pLKOAS3W-hyg+FLuc harboring *firfly luciferase* reporter was from Yi-Ling Lin (IBMS, Academia Sinica, Taiwan). HA-GFP/pLKO_as3w.puro has been described previously [24]. The human angiotensin-converting enzyme II (hACE2) was amplified from human 293T/17 cell cDNA by PCR with the primers 5′-CGGGGTACCACCATGTTCGTGTTTCTGGTCCTG-3′ and 5′-ATGATGACCGGTTCAGGCGTAATCGGGGAC-3′. The wild-type S of SARS-CoV-2 strain Wuhan-Hu-1 (QHD43416.1) from codon-optimized gene synthesis was from Shih-Jen Liu (NHRI, Miaoli, Taiwan). The cDNA of SARS-CoV-2 S was amplified by PCR with the primers 5′-CGGGGTACCACCATGTTCGTGTTTCTGGTCCTG-3′ and 5′-ATGATGACCGGTTCAGGCGTAATCGGGGAC-3′ and cloned to the pcDNA3.1 vector with a C-terminal HA-tag (SARS-CoV-2 S-HA/pcDNA3.1). The SARS-CoV-2 N501Y and D614G mutants were generated by single primer mutagenesis [25] with the primer 5′-ACGGCTTCCAGCCCACATATGGCGTGGGCTATCAGCCT-3′, and 5′-GTGGCCGTGCTGTATCAGGGCGTTAACTGTACCGAGGTGCCTGTGG-3′. The codon-optimized E-2A-M (Figure 1a) and SARS-CoV-2 S variants (Figure 7a) were synthesized by Protech Technology, Taipei, Taiwan.

### 4.3. Cell Lines

Baby hamster kidney BHK-21 cells were cultured in RPMI (SH30027.01, HyClone, Logan, UT, USA) containing 5% fetal bovine serum (FBS). The BHK-21 cells were stably transduced with the lentiviral vector harboring hACE2 and selected with blasticidin (#ant-bl, InvivoGen, San Diego, CA, USA) at 100 μM to obtain BHK-hACE2 cells. Human embryonic kidney 293T/17 cells were grown in DMEM (SH30022.02, HyClone) containing 10% FBS.

### 4.4. Immunofluorescence Assay

BHK-hACE2 cells were fixed with 4% paraformaldehyde in phosphate-buffered saline (PBS; BF203-5L, Protech Technology, Taipei, Taiwan) for 30 min at room temperature. Cell surface non-specific binding was blocked with skim milk in PBS before surface staining with the anti-hACE2 (1:1000) and Alexa Fluor 488-conjugated goat anti-rabbit antibodies (1:1000; A11008, Invitrogen, Rockford, IL, USA). The nuclear counterstain of DAPI (0.25 ng/mL; D1306, Thermo Fisher Scientific, Eugene, OR, USA) was performed before photography using fluorescence microscopy (Olympus IX73).

### 4.5. Western Blot Analysis

Samples were lysed with the RIPA buffer (10 mM Tris, pH 7.5, 5 mM EDTA, 150 mM NaCl, 0.1% SDS, 1% TritonX-100, 1% sodium deoxycholate) including a cocktail of protease inhibitors (04693132001, Roche, Mannheim, Germany) and phosphatase inhibitors (04906837001, Roche). Sample protein concentration was determined by the DC Protein Assay Kit (5000120, BIO-RAD, Hercules, CA, USA). The equivalent amounts of proteins were separated by SDS-PAGE and transferred to a nitrocellulose membrane (XR-IGE-10600003, Amersham, Darmstadt, Germany). Non-specific antibody binding sites were blocked by skim milk in PBS with 0.1% Tween 20 (PBST) then reacted with the indicated primary antibodies followed by appropriate horseradish peroxidase (HRP)-conjugated secondary antibodies (111-035-144 and 115-035-146, Jackson ImmunoResearch, West Grove, PA, USA; A0170, Sigma-Aldrich, Saint Louis, MO, USA). Signals were revealed by Chemiluminescence HRP Substrate (WBKLS0500, Millipore, Darmstadt, Germany) and detected by UVP (ChemiDoc-It Imaging system, Analytik Jena, Germany) using VisionWorks™ LS v8.20 software.

### 4.6. Pseudovirus Propagation

293T/17 cells were co-transfected by Lipofectamine 2000 Transfection Reagent (11668500, Invitrogen) in the presence or absence of the mentioned inhibitors with the following plasmids: helper pCMVΔR8.91 (0.9 μg), reporter pLKOAS3W-hyg+FLuc (1 μg), and the structural protein mentioned (0.1 μg). The pseudotyped lentiviruses were harvested at 24, 36, and 48 h post-transfection. Cell debris in the virus-containing supernatant was removed by low-speed centrifugation followed by filtration with a low protein-binding 0.45-μm pore-size filter (SLHPR33RS, Merck Millipore) at 4 °C. The pseudovirion was quantified by RT-qPCR measuring the viral genomic RNA concentration using the primers targeting Woodchuck Hepatitis Virus Posttranscriptional Regulatory Element (WPRE) (5′-TGTGAAAGATTGACTGGTATTCTTAAC-3′ and 5′-GGAAAGGAGCTGACAGGTGGTG3′) of the genome. The plasmid pLKOAS3W-hyg+FLuc containing the WPRE sequence was used to establish the 10-fold serial dilution standard curve in the qPCR assay.

### 4.7. Transmission Electron Microscope (TEM)

The virions in supernatant were concentrated by Amicon^®^ Ultra 0.5 mL Centrifugal Filters (UFC5100BK, Merck, Kenilworth, NJ, USA). Four microliter supernatant was blotted with the grids through a Hydrophilic Treatment Device (JEOL, HDT-400), then the virions were stained with 2% uranyl acetate and revealed by a JEM1400 electron transmission microscope (JEM-1400, Jeol Ltd., Tokyo, Japan). The virion images were recorded by Ultrascan 4000 4 k × 4 k CCD Camera System Model 895 with an acceleration voltage of 120 KeV.

### 4.8. Flow Cytometry Assay

BHK-hACE2 cells were detached by PBS-EDTA at 72 h post-infection of the pseudovirus harboring GFP reporter. The cells were washed and resuspended in PBS with 0.1% NaN3 and 2% FBS. The cells expressing GFP were revealed and analyzed by BD FACSCalibur and FlowJo VX software.

### 4.9. Binding Assay

BHK-hACE2 cells were incubated with the pseudovirions at 4 °C for 3 h. The unbound pseudovirions were washed away by ice-cold PBS twice. The genomic RNA of the cell-bound pseudovirion was extracted with the total cellular RNA by using an RNeasy Mini Kit (74106, Qiagen, Hilden, Germany). One microgram of total RNA was used for reverse-transcription to cDNA with the High-Capacity cDNA Reverse Transcription Kits (4368814, Applied Biosystems). RT-qPCR measured RNA of the viral genome and cellular actin with specific primer sets (5′-CGACGCAGGTGTCGCAGG-3′ and 5′-GGCGATCTTTCCGCCCTT-3′ for the reporter gene in pseudovirus; 5′-GAAGCATTTGCGGTGGACGAT-3′ and 5′-GCCGCACCATTGGTCTTCTC-3′ for cellular actin). 2X SYBR Green MASTER Mix (4385612, Applied Biosystems) was used for qPCR analysis. The relative binding activity was calculated by analyzing the relative levels of genomic RNA copy numbers normalized to actin.

### 4.10. Neutralization Assay

The pseudovirus was incubated with antibodies at the indicated concentrations to a final volume of 200 μL with serum-free media at 37 °C for 1 h. The mixture was used to infect BHK-hACE2 cells for another 6 h. Then, the cells were washed twice with PBS and replaced with fresh complete culture media. The cell lysates were harvested at 48 h post-infection and analyzed for luciferase activity. The relative infectivity was calculated by the fold to the control group. The NT50 was calculated by Prism 6 (GraphPad). For the NT50 using authentic SARS-CoV-2, the experiments were done by Jian-Jong Liang and Chun-Che Liao at Yi-Ling Lin’s laboratory. Briefly, serially diluted samples were incubated with 100 TCID50 SARS-CoV-2 TCDC#4 (hCoV-19/Taiwan/4/2020) at 37 °C for 1 h. The mixtures were then added to pre-seeded Vero E6 cells for 4-day incubation. Cells were fixed with 10% formaldehyde and stained with 0.5% crystal violate for 20 min. The plates were washed with tap water and scored for infection. The 50% protective titer was calculated by the Reed and Muench Method.

### 4.11. Reporter Assay

The Luciferase Assay System (E1501, Promega, Madison, WI, USA) with the GLOMAX Multi+ Microplate Multimode Reader (Promega) was used to detect luciferase activity.

### 4.12. Quantification and Statistical Analysis

Data are shown as mean ± SD. The difference between two groups was compared by the two-tailed Student’s *t* test. *p* < 0.05 was considered statistically significant.

## Data Availability

All data supporting the conclusions of this study are in the article.
